# On the Virtues of the Spider's Web

**Published:** 1809-09

**Authors:** 


### *To the Editors of the Medical and Physical Journal.*

Gentlemen,

If you rejected my last Communication, because it contained personal and irrevelant observations, I hope this will not meet the same fate, from the same cause.

The high, and I fear, extravagant encomiums, with which Dr. Jackson set forth the sanative powers of the spider's-web, not only in intermittent but in other fevers, would naturally have a tendency to elevate expectation. Being one of those persons who wish to try every thing for the cure or alleviation of disease, that comes fairly recommended, or has a chance of success; and, after reading Dr. Jackson's paper, I immediately set about selecting the most recent cob-webs I could find, in order to exhibit them in an obstinate case of intermittent fever then under my care. I scrupulously attended to the Doctor's directions in making up the pills and in exhibiting them, but held out no extraordinary expectations to the patient, nor informed her what she was taking. To my own, as well as the patient's disappointment, the rigor came on and went through its course as usual, succeeded by the same degree of fever; nor could I discover, by a strict examination, that the patient had felt any alteration whatever in regard to her sensations. The pills were, in this case, evidently inert and useless. About the same time, a neighbouring surgeon was induced, from Dr. Jackson's report, to try the cob-web also; the result was, (as he informed me) that the disease was not at all arrested in its progress, nor had the pills any discoverable effect. How these apparent inconsistences, in regard to Dr. Jackson's report and mine are to be reconciled, I cannot tell? You suggest a probability, that the webs which Dr. Jackson employed, were produced from a different species of spider: it may be so; though I think, that in the numerous cases in which Dr. J. appears to have given them, he would have met with the different kinds. An error must exist somewhere, perhaps Dr. J. will be able to throw more light light upon the subject; for surely, if there are cob-webs that possess such important medicinal powers as Dr. Jackson has described, the medical profession would be extremely culpable in passing by the subject without further investigation.

A Correspondent of yours, a Mr. Scott, is perfectly right in conjecturing me to be the writer of the communication upon "Arsenic in Intermittent Fevers." I think every practitioner would agree with me in disapproving of female interlopers, especially if they hesitate not to exhibit such a potent and poisonous article as arsenic. Who would not expect to see mischief? Upon further consideration, surely Mr. Scott will "*altogether disapprove*" of such bold and inconsiderate practice, though, at present, he seems to entertain a high notion of the abilities of "*Lady Doctors*," more especially in "*simple complaints*."

I agree decidedly with Mr. S. that a timid and uncertain exhibition of our most useful medicines is too often met with; but this extreme, in my opinion, is quite as safe to the patient as the contrary, and much less hazardous to the credit and reputation of the practitioner. Mr. Scott's situation is widely different from that of a country surgeon; he may exhibit his medicines in what quantity and form he pleases with impunity; and the constitution of a sailor will hardly bear a comparison with that of a weak delicate female. But a country practitioner is often watched with unceasing attention; patients are not easily persuaded that a medicine should act violently in order to do good; or, if he commits a mistake, it may be detected, and is not soon forgotten, perhaps he is discarded; the patient has influence with friends; and there are persons who would dwell, with studied aggravation, upon the error, in order to shake and ruin the confidence of the patient in the practitioner. This is by no means an uncommon case, and is more especially met with by the younger branches of the profession.

Mr. Scott's case will go but little way to corroborate Dr. Jackson's testimony in favour of the cob-web. The abhorence and fear excited in the lady by swallowing the spider, will sufficiently account for the disappearance of the rigor. The operation of fear upon the system will produce even death itself; and a much less degree of it, would be sufficient to cure the paroxysm of an ague.

The indiscriminate manner in which Mr. Scott condemns all anonymous publications, surprises me extremely; if he had only been a constant reader of your pages, pages, he must have met with many anonymous papers, replete with science and knowledge. A man may have facts of the greatest importance to communicate, and yet may have many reasons for with-holding his name; situated as he is, there can scarcely be a motive or a wish for concealment, but situated as I am, I must once more subscribe myself,

AN ESSEX PRACTITIONER.

*August* 4, 1809.

---

I have subjoined a Case, and leave you to decide upon the propriety of inserting or rejecting it. It furnishes no practical results of importance, but is, I think, sufficiently extraordinary to deserve recording.

JAMES CLAYTON, aged 64, a healthy strong man, was admitted into Guy's Hospital, in the spring of 1808, under the care of Mr. Cooper, for a disease of the scrotum. He gave the following account of himself and of the origin of the complaint. In 1792, he was a private in the expedition to Holland, under the command of the Duke of York, and being exposed in the retreat of the army to severe cold, his scrotum was frozen; the part according to his description first became white, and soon changed to red and afterwards black; at the end of a fortnight the scrotum burst, and great part of it separated so as to expose and lay bare the testes; at the commencement of the complaint he was obliged to march on foot, but soon after the separation of the parts, he was carried upon a waggon, and after marching and riding for near a month, he found temporary quarters in a barn; during his stay here, the wound healed, the testicles being covered, as he said, with new skin; this took place about two months from the separation of the scrotum; during this period, the only application made use of was a poultice composed of bread, lard, and water; the discharge was but small in quantity; he remarked that the scrotum and penis afterwards continued much enlarged.

After landing in England, he was discharged, and laboured as a husbandman for his livelihood, without inconvenience from his former complaint, till about Christmas, 1807, fifteen years after his return from Holland, when being much exposed to wet and cold in his occupation, the scrotum began to look red and swell, with much pain; this swelling increased and became larger than his two fists, and after suffering much pain for three weeks, the principal part of the scrotum separated and again laid the testicles cles bare. The man now sent for medical advice, and the surgeon to whom he applied sent him to Guy's Hospital, at which time he was in the following state.—His health was good, and his constitution appeared pretty strong. The line of separation was in a healthy granulating state; the left testicle was larger than the right, and almost completely exposed; the right testicle was smaller and not so much exposed. The bulb of the penis was seen covered partly by the accelerator urinæ muscle; the testes were but slightly sensible, the man not complaining upon their being gently pressed or handled; the penis was very large and flaccid. The man continued for several weeks in the hospital, during which time the granulating process gradually proceeded; nothing material was done on account of his obstinacy. Constitutional symptoms of disease now appeared; he complained of shortness of breath, with pain and uneasiness in the chest, and a cough; some medicines were ordered, but these symptoms increased, and he soon after died, as supposed, of Hydrothorax. The unusual confinement in the air of an hospital, probably, assisted in bringing on and hastening his death.

It was Mr. Cooper's wish to remove the left testicle, and then to bring the separated edges of the sore together by sutures, and this could not be done unless the testicle was removed; but the man would not consent to the operation though he was repeatedly solicited and advised. As no other probable assistance could be afforded, the parts were left to nature, and no doubt the wound would again have healed by granulation if the man had lived.

I regret I cannot send you a sketch of the parts. Mr. Cooper pointed out the singularity of the appearance repeatedly to the pupils, and, if I mistake not, his assistant made a drawing of it.

